# Interleukin-38 Ameliorates Atherosclerosis by Inhibiting Macrophage M1-like Polarization and Apoptosis

**DOI:** 10.3390/biom15121741

**Published:** 2025-12-16

**Authors:** Zhiyang Li, Xuelian Li, Rui Shen, Yue Wang, Jian Yu, Chengliang Pan, Yifan Cai, Qian Dong, Kunwu Yu, Qiutang Zeng

**Affiliations:** 1 Department of Cardiology, Union Hospital, Tongji Medical College, Huazhong University of Science and Technology, Wuhan 430022, China; d202482317@hust.edu.cn (Z.L.);; 2 Hubei Key Laboratory of Biological Targeted Therapy, Union Hospital, Tongji Medical College, Huazhong University of Science and Technology, Wuhan 430022, China; 3 Hubei Provincial Engineering Research Center of Immunological Diagnosis and Therapy for Cardiovascular Diseases, Union Hospital, Tongji Medical College, Huazhong University of Science and Technology, Wuhan 430022, China; 4 Key Laboratory of Biological Targeted Therapy, Ministry of Education, Huazhong University of Science and Technology, Wuhan 430022, China

**Keywords:** atherosclerosis, inflammation, macrophage, Interleukin-38

## Abstract

Objectives: As a novel member of the interleukin(IL)-1 family, IL-38 has shown therapeutic effects in various chronic inflammatory diseases. However, its role and underlying mechanisms in cardiovascular diseases, particularly atherosclerosis, remain unclear. This study aimed to explore the effects of IL-38 on atherosclerosis progression and its mechanisms in regulating macrophage function during the atherosclerotic process. Methods: To evaluate the therapeutic potential of IL-38 in atherosclerosis, we performed histopathological examinations and biochemical analyses in vivo. In vitro, we used primary bone marrow-derived macrophages (BMDMs) stimulated with oxidized low-density lipoprotein (ox-LDL) to assess the anti-inflammatory effects of IL-38 and quantified its impact on ox-LDL-induced macrophage polarization. To further elucidate the specific mechanisms by which IL-38 regulates macrophage function, we conducted mRNA sequencing and validated downstream regulatory signaling pathways. Results: IL-38 exhibited therapeutic potential in atherosclerosis by reducing atherosclerotic plaque formation, modulating plaque composition, suppressing the production of proinflammatory cytokines within plaques, and potentially regulating macrophage cholesterol metabolism. Moreover, IL-38 exerted significant anti-inflammatory effects on macrophages both in vivo and in vitro. Notably, it inhibited the polarization of macrophages toward the proinflammatory M1-like phenotype in both settings. Additionally, IL-38 impeded the phosphorylation and nuclear translocation of p65 in BMDMs and reduced ox-LDL-induced macrophage apoptosis. Conclusion: IL-38 holds therapeutic potential for atherosclerosis, as it alleviates disease progression, inhibits macrophage polarization toward the M1-like phenotype, suppresses nuclear factor-κB (NF-κB) signaling activation, and reduces macrophage apoptosis. This study provides new insights into the anti-inflammatory mechanisms by which IL-38 mitigates atherosclerosis.

## 1. Introduction

Atherosclerosis is a complex, chronic immune-inflammatory disorder that primarily affects large- and medium-sized arteries, with its pathogenesis largely driven by lipid components circulating in the bloodstream [1]. Abnormal lipid deposition in the arterial endothelium triggers excessive production of oxidized low-density lipoprotein (ox-LDL), which in turn initiates the differentiation and recruitment of inflammatory cells, as well as the secretion of various cytokines and inflammatory mediators [2,3]. Mature macrophages phagocytosing ox-LDL differentiate into foam cells. Subsequent death of these foam cells forms lipid-rich necrotic cores, which are overlaid with a fibrous cap and ultimately lead to atherosclerotic plaque development. When persistent inflammatory factors disrupt the integrity of the plaque’s fibrous cap, this disruption induces blood flow turbulence, promotes thrombus formation, impairs distal blood perfusion, and ultimately precipitates adverse cardiovascular events [4,5].

Macrophages, as a core component of innate immunity, play a pivotal role in the inflammatory cascade of atherosclerosis. Throughout the pathogenesis of atherosclerosis, macrophages make substantial contributions to disease progression, not only via initial lipid uptake but also through the diverse cell phenotypes they adopt and the array of cytokines they secrete [6,7]. Traditionally, macrophages are classified based on their functional properties into classically activated macrophages (M1-like) and alternatively activated macrophages (M2-like) [8]. M1-like macrophages are primarily responsible for initiating and amplifying inflammatory responses, whereas M2-like macrophages are predominantly involved in tissue repair and regeneration [9]. Notably, previous studies have demonstrated that dysregulation of intracellular lipids, metabolites, and proinflammatory mediators can modulate macrophage polarization [7]; furthermore, effective regulation of macrophage phenotype switching has been shown to attenuate the progression of atherosclerosis [10]. Although recent research has revealed that macrophage phenotypes in atherosclerosis are not restricted to the binary M1/M2 paradigm, rather encompass a spectrum of intermediate or specialized phenotypes [11,12], modulating macrophages toward a tissue-reparative phenotype and suppressing excessive inflammation remain important potential therapeutic targets for atherosclerosis [13]. Meanwhile, following macrophage foam cell transformation (a process driven by excessive uptake of oxidized low-density lipoprotein), macrophage apoptosis and necrosis are further triggered. The release of cellular debris and intracellular contents from dying macrophages acts as a novel proinflammatory stimulus, which activates surrounding immune cells, amplifies the inflammatory response, and thereby exacerbates atherosclerotic plaque formation [14]. Therefore, inhibiting macrophage apoptosis during atherosclerosis progression represents a viable strategy for ameliorating pathological plaque development [13,15,16].

Interleukin-38 (IL-38), a novel member of the interleukin-1 (IL-1) superfamily, is broadly expressed in multiple human immune tissues and organs, with the highest expression levels detected in proliferating B cells isolated from skin and tonsils [17,18,19,20,21]. A growing body of evidence has highlighted its potential therapeutic value as an anti-inflammatory cytokine in metabolic disorders and cardiovascular diseases [21]. Our previous clinical study also demonstrated a correlation between IL-38 levels and the progression of myocardial infarction [22]. However, despite its established association with myocardial infarction, an acute cardiovascular event, the specific role and underlying mechanism of IL-38 in the development of atherosclerosis, a chronic inflammatory disease, remain unclear [23].

In this study, we investigated the effects of exogenous IL-38 on macrophages in the context of murine atherosclerosis, employing a combination of in vivo and in vitro experimental approaches. Our findings suggest that IL-38 exerts a potential inhibitory effect on the progression of atherosclerosis in mice by suppressing M1-like macrophage polarization and apoptosis, as well as attenuating the activation of the nuclear factor-κB (NF-κB) signaling pathway.

## 2. Methods

### 2.1. Animals

ApoE^−/−^ male mice on a C57BL/6J background and wild-type (WT) C57BL/6J mice were purchased from Beijing Vital River Laboratory Animal Technology Co. Ltd. (Beijing, China). All mice were housed in a specific pathogen-free facility (Tongji Medical College, Wuhan, China) and provided with a standard chow diet. To establish an atherosclerotic model, 8-week-old ApoE^−/−^ mice were fed a Western diet for 12 consecutive weeks. For the control group, mice were administered intraperitoneal injections of 200 μL phosphate-buffered saline (PBS) twice weekly, whereas the experimental group received 200 μL of PBS solution containing 1 μg of IL-38 (AdipoGen, San Diego, CA, USA) twice weekly via intraperitoneal injection [24,25,26]. The injection duration was consistent with the Western diet feeding period (12 weeks), and no anesthetics were used during the injection procedures. At the end of the experiment, mice were euthanized by cervical dislocation following standard procedures. All experimental protocols were performed in strict compliance with the guidelines of the Institutional Animal Care and Use Committee of Tongji Medical College, Huazhong University of Science and Technology, and have been approved by the university’s ethical review board.

### 2.2. Cell Culture

Bone marrow-derived macrophages (BMDMs) were isolated and cultured from wild-type C57BL/6J mice. Briefly, fresh bone marrow was flushed from the femurs and tibias. After lysis of erythrocytes using erythrocyte lysis buffer and subsequent washing with phosphate-buffered saline (PBS), the remaining cells were cultured in high-glucose Dulbecco’s Modified Eagle Medium (DMEM) supplemented with 10% heat-inactivated fetal bovine serum (FBS) and 20 ng/mL macrophage colony stimulating factor (M-CSF; MedChemExpress Co., Ltd., Monmouth Junction, NJ, USA) for 7 consecutive days to induce macrophage differentiation. To assess the purity of BMDMs, cells were stained with fluorochrome-conjugated antibodies against CD11b (anti-CD11b-PerCP-Cyanine5.5; eBiosciences, San Diego, CA, USA) and F4/80 (anti-F4/80-PE; eBiosciences, USA), followed by analysis via flow cytometry to determine macrophage purity (Figure S1). For the macrophage foam cell formation assay, BMDMs were incubated with 50 μg/mL oxidized low-density lipoprotein (ox-LDL; Yiyuan Biotec, Guangzhou, China) under two experimental conditions: in the presence or absence of IL-38. To investigate the regulatory effect of IL-38 on the NF-κB signaling pathway, BMDMs were co-treated with 50 μg/mL ox-LDL, 50 ng/mL IL-38 (Figure S2C), and an additional 30 ng/mL NF-κB activator 1 (MedChemExpress Co., Ltd., Monmouth Junction, NJ, USA).

### 2.3. Body Weights and Plasma Lipid Levels

Body weight of the mice was measured once weekly starting from the initiation of the Western diet. For the determination of serum cholesterol and triglyceride levels, Whole blood was collected and allowed to coagulate at room temperature for 30–60 min. Subsequently, serum was separated by centrifugation at 1000× *g* for 15 min. Total cholesterol (TC), triglycerides (TG), low-density lipoprotein cholesterol (LDL-C) and high-density lipoprotein cholesterol (HDL-C) concentrations were quantified using enzymatic methods on an automatic biochemical analyzer (Hitachi, Tokyo, Japan).

### 2.4. Oil Red O Staining and Quantification

Hearts and aortas were carefully harvested from mice and gently rinsed with PBS to remove residual blood and debris. The aorta was then longitudinally opened along its long axis to prepare for en face analysis. For histological examination of the aortic root, the heart with attached aortic root was fixed in 4% paraformaldehyde (PFA) at 4 °C for 24 h, followed by embedding in Optimal Cutting Temperature (OTC) embedding compound. Serial cryosections (7 μm thick) were cut through the aortic sinus region. Concurrently, glass coverslips with adherent macrophages were fixed in 4% PFA for 15 min at room temperature to preserve cellular morphology. Aortic en face preparations, aortic root sections, and macrophage coverslips were stained with filtered Oil Red O solution (Sigma-Aldrich, St. Louis, MO, USA) to visualize lipid accumulation. After staining, samples were thoroughly rinsed with distilled water to remove excess dye, differentiated briefly with 70% ethanol to reduce background staining, and air-dried at room temperature. Stained samples were mounted with glycerol gelatin and examined under a light microscope (Olympus, Tokyo, Japan). ImageJ 1.53a (National Institutes of Health, Bethesda, MD, USA) was employed for quantification of aortic sinus plaque area and aortic en face plaque coverage.

### 2.5. Immunohistochemistry and Immunofluorescence

After 12 weeks on a Western diet, mice were euthanized. The heart-aortic junction (heart attached to the aortic root) was fixed, embedded in paraffin wax, and cut into 3 μm serial sections. We stained CD31, CD68, α-SMA, and Masson’s trichrome staining of the aortic sinus to identify endothelial cells, macrophages, smooth muscle cells, and collagen fibers, respectively. Anti-CD31, anti-CD68 and anti-α-SMA antibodies were obtained from Abcam (Cambridge, UK). Masson’s Trichrome Stain Kit was purchased from Beyotime Biotechnology (Shanghai, China). Counting and analyzing were performed using ImageJ. For immunofluorescence, aortic arch sections were double-stained to detect macrophage subsets: mouse anti-CD68 (pan-macrophage marker) and mouse anti-CD86 (M1-like marker) for M1-like macrophages, and mouse anti-CD68 and mouse anti-CD206 (M2-like marker) for M2-like macrophages (all antibodies from Abcam, Cambridge, UK). Terminal deoxynucleotidyl transferase-mediated dUTP nick end labeling (TUNEL) assay was performed using a commercial kit (Roche, Basel, Switzerland) following the manufacturer’s protocol. To assess p65 expression and subcellular localization in BMDMs, immunostaining was conducted with rabbit polyclonal anti-p65 (Abcam, USA). All sections were counterstained with hematoxylin, and quantification was performed using ImageJ software.

### 2.6. Western Blot Analysis

Protein was extracted from the atherosclerotic plaque and BMDMs. The concentration was determined using a Bio-Rad protein assay kit (Bio-Rad Laboratories, Inc., Hercules, CA, USA). To block the membranes, 5% defatted milk was used. The blots were then incubated with primary antibodies and horseradish peroxidase (HRP)-conjugated secondary antibodies. The primary antibodies, including anti-ATP binding cassette transporter G1 (ABCG1), anti-ATP binding cassette transporter A1 (ABCA1), anti-scavenger receptor A (SR-A), anti-B-cell lymphoma-2 (Bcl2), anti-Bcl2-associated X protein (Bax) and anti-β-actin, were obtained from Proteintech (Wuhan, China), while anti-p65 and anti-Phospho-p65 antibodies were sourced from Abcam (USA). All secondary antibodies were also from Proteintech (Wuhan, China). The specific bands were visualized using the Super enhanced chemiluminescence (ECL) substrate (Nanjing Vazyme Biotech Co., Ltd., Nanjing, China). Images were captured and analyzed with Image Lab 3.0 software (Bio-Rad Laboratories, Inc., Hercules, CA, USA). Original Western blots can be found in Supplementary Materials.

### 2.7. Flow Cytometry

Cells from spleen and BMDMs were incubated for 30 min with a panel of antibodies: Anti-CD45-APC-Cy7, anti-CD11b-PerCP-Cyanine5.5, Anti-F4/80-PE, anti-CD86-PE-Cy7, and Anti-CD206-APC, all of which were sourced from eBioscience (San Diego, CA, USA). Subsequently, flow cytometry analysis was conducted using a FACSCalibur system (BD Immunocytometry Systems, San Jose, CA, USA), and the resultant data were processed with FlowJo V10 software. Additionally, the rate of apoptosis was assessed using a fluorescein isothiocyanate (FITC)-labeled Annexin V Apoptosis Detection Kit (BD Biosciences, San Jose, CA, USA).

### 2.8. Real-Time PCR

Total RNA was extracted from BMDMs using a Trizol-like RNA isolation reagent (Nanjing Vazyme Biotech Co., Ltd., Nanjing, China) according to the manufacturer’s protocol. The quality of the RNA was evaluated based on the A260/A280 and A260/230 ratios. cDNA was synthesized using HiScript III RT SuperMix for qPCR (Nanjing Vazyme Biotech Co., Ltd., Nanjing, China). Real-time PCR was performed using SYBR Green Master Mix (Nanjing Vazyme Biotech Co., Ltd., Nanjing, China) on a CFX Connect Real-Time PCR Detection System. The specific primers used for real-time PCR in this study are detailed in Table S1.

### 2.9. mRNA Sequencing

#### 2.9.1. RNA Isolation and Library Preparation

Using the TRIzol reagent from Invitrogen (USA), total RNA was extracted in adherence to the manufacturer’s protocol. The purity and concentration of the RNA were then assessed using the NanoDrop 2000 spectrophotometer (Thermo Scientific, Waltham, MA, USA). To evaluate RNA integrity, the Agilent 2100 Bioanalyzer (Agilent Technologies, Inc., Santa Clara, CA, USA) was employed. Subsequently, RNA libraries were constructed utilizing the VAHTS Universal V6 RNA-seq Library Prep Kit, following the manufacturer’s instructions. The transcriptome sequencing and analysis were carried out by OE Biotech Co., Ltd. (Shanghai, China).

#### 2.9.2. mRNA Sequencing Analysis Process: RNA Sequencing and Differentially Expressed Genes Analysis

The sequencing of the libraries was conducted on an Illumina Novaseq 6000 platform (Illumina, Inc., San Diego, CA, USA), generating 150 bp paired-end reads. The raw reads in fastq format were initially processed through fastp, where low-quality reads were removed to yield clean reads. Approximately 47 clean reads per sample were retained for further analysis. These clean reads were then mapped to the reference genome using HISAT. The FPKM (Fragments Per Kilobase of transcript per Million mapped reads) values for each gene were calculated, and the read counts were obtained using HTSeq-count. Principal Component Analysis (PCA) was carried out using R (v 3.2.0) to assess the biological replication of the samples.

For differential expression analysis, DESeq2 was utilized. A threshold of Q value < 0.05 and fold change > 2 or fold change < 0.5 was set to identify significantly differentially expressed genes (DEGs). Hierarchical cluster analysis of the DEGs was performed using R (v 3.2.0) to illustrate the gene expression patterns across different groups and samples. A radar map of the top 30 genes was created using the R 4.3.2 (R Foundation for Statistical Computing, Vienna, Austria) ggradar to visualize the expression of up-regulated and down-regulated DEGs.

Kyoto Encyclopedia of Genes and Genomes (KEGG) pathway enrichment analyses of the DEGs were conducted based on the hypergeometric distribution. R (v 3.2.0) was used to screen for significant enriched terms and to create visual representations of these terms.

Gene Set Enrichment Analysis (GSEA) was performed using GSEA 4.3.2 (Broad Institute of MIT and Harvard, Cambridge, MA, USA). This analysis utilized a predefined gene set, with genes ranked according to their degree of differential expression between the two types of samples. The analysis aimed to determine whether the predefined gene set was enriched at the top or bottom of the ranking list.

### 2.10. Enzyme-Linked Immunosorbent Assay

The concentration of plasma IL-38 in experimental samples was quantified using ELISA kits. Plasma samples isolated from WT and ApoE^−/−^ mice were assayed with the Mlbio ELISA kit (catalog no. mlC50322-2661) strictly following the manufacturer’s standardized protocol. Absorbance values were measured at a wavelength of 450 nm, and the IL-38 concentration in each sample was calculated by interpolating against a standard curve generated in parallel with the samples.

### 2.11. Statistics

All data were expressed as mean ± SEM. For comparisons between two groups, Student’s *t*-test was employed when the data adhered to a normal distribution and the variances between groups were equivalent. In instances where the data deviated from a normal distribution or the group variances were unequal, the Mann–Whitney rank sum test was utilized. When conducting multiple comparisons among three or more groups, one-way ANOVA was applied, followed by the Holm–Sidak test for normally distributed data with equal group variances. Conversely, for data that were not normally distributed or had unequal variances, the Kruskal–Wallis test was used, with subsequent analysis by the Dunn test. Statistical significance was determined at *p* < 0.05.

## 3. Results

### 3.1. IL-38 Alleviates Atherosclerosis and Modulates Plaque Composition

**Figure 1:**
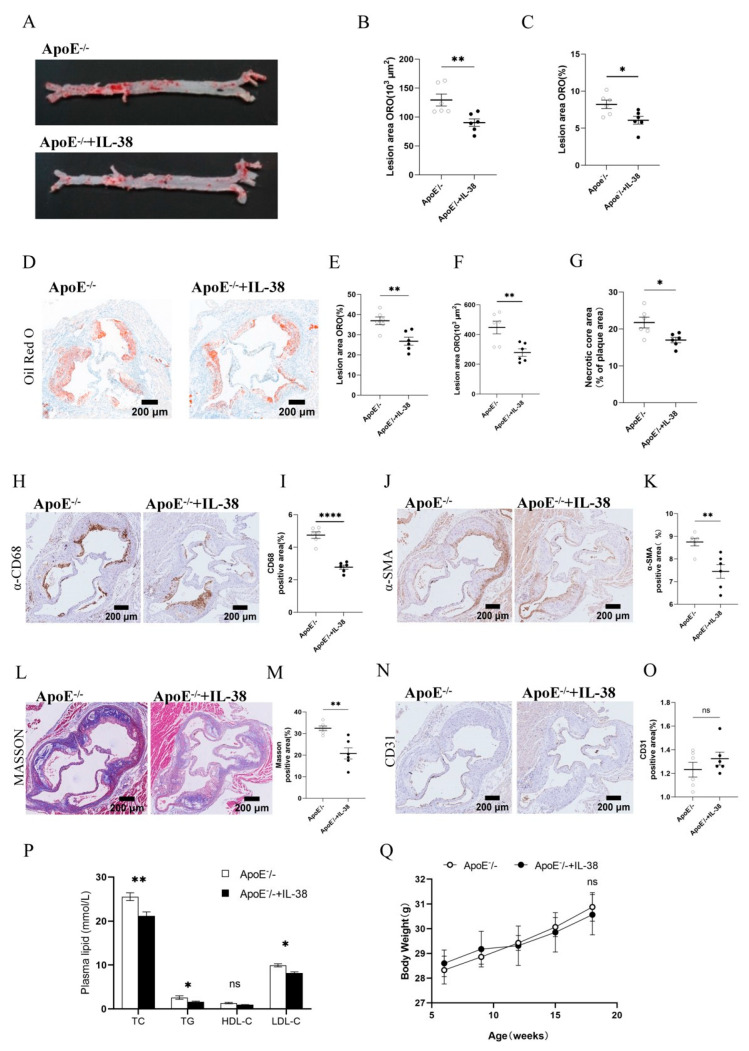
IL-38 administration affects atherosclerotic plaque formation and composition in ApoE^−/−^. mice fed a Western diet for 12 weeks. (**A**) Oil Red O en face staining of whole aortas. (**B**) Quantitative analysis of total atherosclerotic plaque area in whole aortas. (**C**) Ratio of total plaque area to whole aorta area. (**D**) Oil Red O staining of aortic sinus sections. (**E**) Ratio of atherosclerotic plaque area to total aortic sinus area. (**F**) Quantitative analysis of atherosclerotic plaque area in aortic sinus sections. (**G**) Ratio of necrotic core area to total plaque area in aortic sinus sections. (**H**) IHC staining of CD68 in aortic plaques. (**I**) Quantitative analysis of CD68-positive area percentage. (**J**) Representative IHC staining of α-SMA in aortic plaques. (**K**) Quantitative analysis of α-SMA-positive area percentage. (**L**) Representative Masson’s trichrome staining of collagen fibers in aortic plaques. (**M**) Quantitative analysis of collagen-positive area percentage. (**N**) Representative IHC staining of CD31 in aortic plaques. (**O**) Quantitative analysis of CD31-positive area percentage. (**P**) Serum levels of TC, TG, HDL-C, and LDL-C measured by enzymatic method. (**Q**) Body weight change curve of mice. *n* = 6 per group. * *p* < 0.05, ** *p* < 0.01,**** *p* < 0.0001, ns, not significant. Error bars represent mean ± SEM.

To investigate the effect of IL-38 on atherosclerotic progression, ApoE^−/−^ mice were administered intraperitoneal injections of exogenous recombinant IL-38. Firstly, we excluded the impact of ApoE gene knockout on IL-38 expression in the peripheral blood and arteries of mice (Figure S2A,B). Following 12 weeks of Western diet feeding, quantitative analysis showed that IL-38 significantly reduced the size of atherosclerotic plaques in the aorta (Figure 1A,B). Consistently, the ratio of total atherosclerotic plaque area to the entire aortic surface area also exhibited a statistically significant decrease (Figure 1C). Additionally, the plaque area, plaque ratio in the aortic sinus, and the proportion of necrotic cores in plaques were all significantly reduced (Figure 1D–G). These results indicate that IL-38 exerts an inhibitory effect on atherosclerotic plaque formation in mice. To further explore the role of IL-38 in regulating plaque composition, we analyzed the key cellular and structural components of plaques. The percentage of CD68^+^ macrophages (Figure 1H,I), α-SMA^+^ vascular smooth muscle cells (Figure 1J,K), and collagen fiber content (Figure 1L,M) in plaques were all significantly reduced in the IL-38 treatment group compared with the control group. However, the percentage of CD31^+^ endothelial cells showed no statistically significant difference between groups (Figure 1N,O). Biochemical analysis of plasma lipids revealed that IL-38 treatment significantly decreased plasma levels of TC, TG, and LDL-C, while no significant difference was observed in HDL-C levels (Figure 1P). There was also no significant difference in body weight among all groups (Figure 1Q). By conducting a correlation analysis of TG, TC and LDL with plaque area, necrotic core area and CD68^+^ area, it can be observed that IL-38 does not improve atherosclerosis by regulating lipid levels (Figure S3).

### 3.2. IL-38 Reduces the Formation of Macrophage-Derived Foam Cells and the Expression of Related Inflammatory Factors

**Figure 2:**
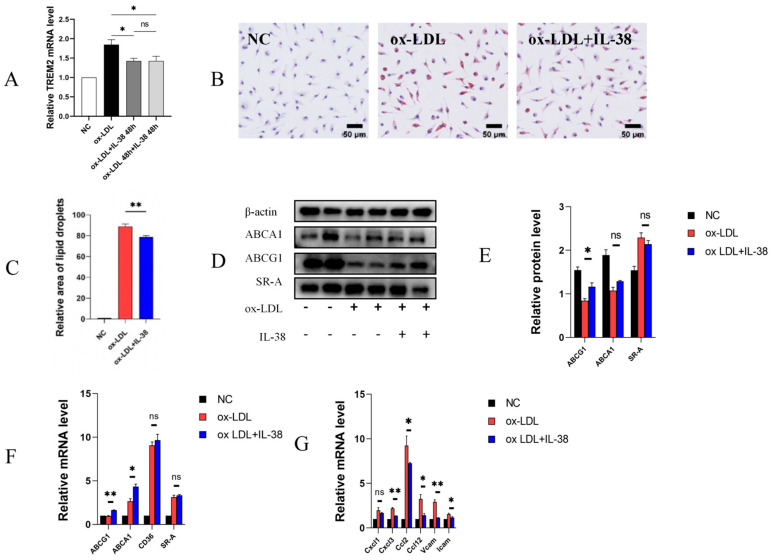
IL-38 affects macrophage cholesterol loading and the expression of inflammatory factors. (**A**) Relative mRNA expression of *TREM2* in macrophages. (**B**) Representative Oil Red O staining of lipid droplets in macrophages. (**C**) Quantitative analysis of Oil Red O-positive area percentage. (**D**) Western blot of ABCA1, ABCG1 and SR-A in macrophages. (**E**) Quantitative analysis of protein expression levels. (**F**) Relative mRNA expression of *ABCA1*, *ABCG1*, *SR-A*, and *CD36* in macrophages. (**G**) Relative mRNA expression of inflammatory cytokines and chemokines in macrophages. *n* = 6 per group. * *p* < 0.05, ** *p* < 0.01, ns, not significant. Error bars represent mean ± SEM.

To further investigate the cellular mechanism by which IL-38 regulates atherosclerosis, we established an in vitro model of macrophage foam cell formation by stimulating BMDMs with ox-LDL. We found that *Trem2* mRNA expression was significantly reduced following IL-38 treatment. However, there was no significant difference in *Trem2* mRNA levels regardless of whether IL-38 was administered concurrently with or after ox-LDL stimulation (Figure 2A). Oil Red O staining revealed a significant reduction in intracellular lipid droplet accumulation in BMDMs treated with IL-38 compared to ox-LDL alone (Figure 2B,C). Given that foam cell formation primarily results from increased ox-LDL uptake and impaired cholesterol efflux in macrophages [7], we next examined the expression of key cholesterol transporters [27] via Western blot (WB) (Figure 2D,E) and quantitative real-time PCR (qPCR) (Figure 2F). Ox-LDL decreases the protein expression of ABCG1 and ABCA1 in macrophages, while increasing the protein expression level of SR-A. After co-stimulating macrophages with IL-38 and ox-LDL, we can observe an increase in the expression of ABCG1, a cholesterol efflux receptor in macrophages. At the transcriptional level, we observed that IL-38 increases the mRNA expression of *ABCG1* and *ABCA1* in macrophages stimulated with ox-LDL. However, the discrepancies observed between the transcriptional and translational levels were likely due to post-transcriptional regulation of mRNA or post-translational modifications of proteins, which may uncouple the abundance of transcripts and proteins. Recent studies have shown that IL-38 alleviates hepatic steatosis by suppressing lipogenic protein expression and endoplasmic reticulum stress markers, thereby reducing lipid accumulation [28], which is consistent with the experimental results of our study. Furthermore, we performed qPCR experiments to assess the expression of inflammatory factors. We further assessed inflammatory factor expression via qPCR. Ox-LDL stimulation significantly increased the mRNA levels of C-C motif chemokine ligand 2 (*Ccl2*), *Ccl12*, C-X-C motif chemokine ligand 1 (*Cxcl1*), *Cxcl3*, intercellular adhesion molecule 1 (*Icam1*), and vascular cell adhesion molecule 1 (*Vcam1*) compared to controls (Figure 2G). Collectively, these results indicate that IL-38 modulates both cholesterol transport and inflammatory responses in ox-LDL-stimulated macrophages, contributing to its anti-atherosclerotic effect.

### 3.3. IL-38 Mitigates Systemic and Atherosclerotic Lesion Inflammation and Decreases M1-like Macrophage Polarization

**Figure 3:**
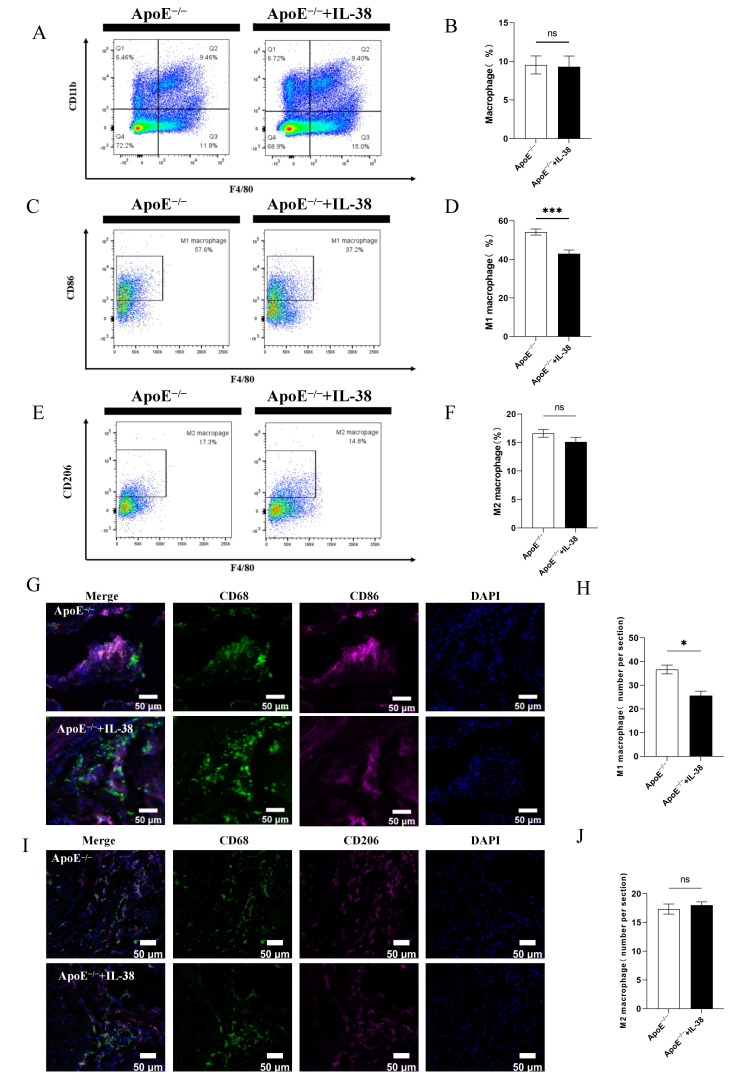
IL-38 affects macrophage M1-like polarization in mouse spleen and bone marrow-derived macrophages. (**A**) Representative flow cytometry dot plots of CD45^+^CD11b^+^F4/80^+^ total macrophages in mouse spleen. (**B**) Quantitative analysis of the proportion of CD45^+^CD11b^+^F4/80^+^ macrophages in splenic immune cells. (**C**) Flow cytometry dot plots of CD45^+^CD11b^+^F4/80^+^CD86^+^ M1-like macrophages in mouse spleen. (**D**) Quantitative analysis of the proportion of splenic CD45^+^CD11b^+^F4/80^+^ CD86^+^ M1-like macrophages. (**E**) Flow cytometry dot plots of CD45^+^CD11b^+^ F4/80^+^CD206^+^ M2-like macrophages in mouse spleen. (**F**) Quantitative analysis of the proportion of splenic CD45^+^CD11b^+^F4/80^+^CD206^+^ M2-like macrophages. (**G**) Representative immunofluorescence images of CD68^+^CD86^+^ M1-like macrophages in atherosclerotic plaques. (**H**) Quantitative assessment of CD68^+^CD86^+^ M1-like macrophages per plaque section. (**I**) Representative immunofluorescence images of CD68^+^CD206^+^ M2-like macrophages in atherosclerotic plaques. (**J**) Quantitative assessment of CD68^+^CD206^+^ M2-like macrophages per plaque section. *n* = 6 per group. * *p* < 0.05, *** *p* < 0.001, ns, not significant. Error bars represent mean ± SEM.

To investigate changes in macrophage populations within the systemic immune response under IL-38 stimulation, we performed flow cytometry analysis on immune cells isolated from mouse spleens. No statistically significant change was observed in the proportion of total macrophages (CD45^+^CD11b^+^F4/80^+^) among splenic immune cells following IL-38 treatment (Figure 3A,B). However, a notable reduction was detected in the proportion of splenic macrophages that differentiated into CD45^+^CD11b^+^F4/80^+^CD86^+^ M1-like macrophages (Figure 3C,D). Conversely, IL-38 did not exert any significant influence on the differentiation of macrophages into CD45^+^CD11b^+^F4/80^+^CD206^+^ M2-like macrophages (Figure 3E,F). To further confirm this phenotypic shift in the atherosclerotic microenvironment, we performed immunofluorescence double staining on atherosclerotic plaques. Consistent with the splenic flow cytometry results, we observed a significant decrease in the proportion of CD68^+^CD86^+^ M1-like macrophages within atherosclerotic plaques (Figure 3G,H), whereas the proportion of CD68^+^CD206^+^ M2-like macrophages exhibited no statistically significant change (Figure 3I,J).

### 3.4. IL-38 Diminishes the Polarization of Macrophages Towards M1-like Phenotype

**Figure 4:**
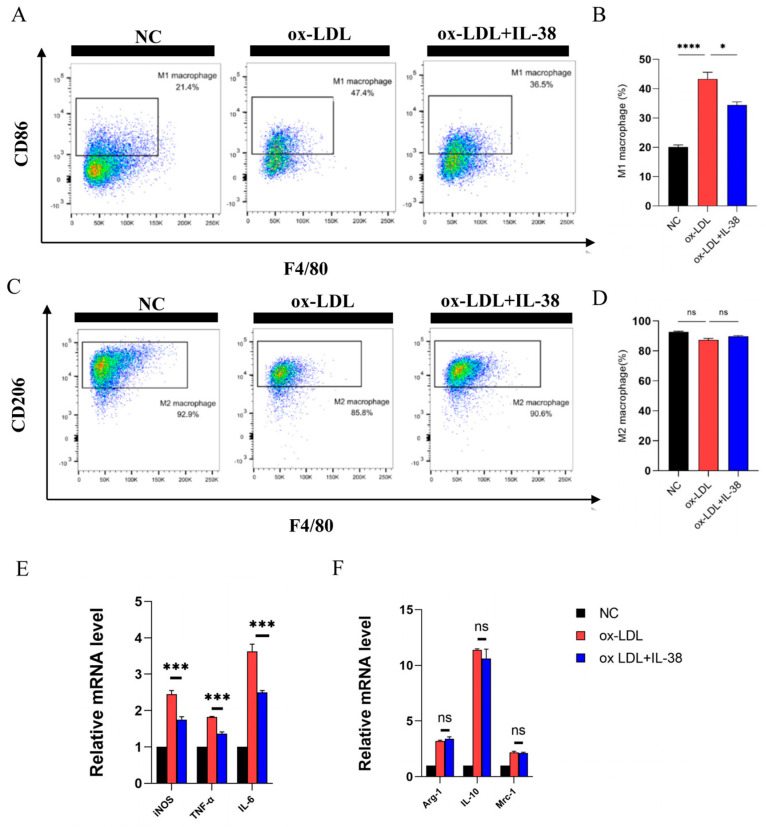
IL-38 affects M1-like macrophage polarization in ox-LDL-stimulated BMDMs. (**A**) Flow cytometry dot plots of CD45^+^ CD11b^+^F4/80^+^CD86^+^ M1-like macrophages in BMDMs. (**B**) Representative flow cytometry dot plots of CD45^+^CD11b^+^F4/80^+^ CD206^+^ M2-like macrophages in BMDMs. (**C**) Quantitative analysis of the proportion of CD45^+^CD11b^+^F4/80^+^CD86^+^ M1-like macrophages. (**D**) Quantitative analysis of the proportion of CD45^+^CD11b^+^F4/80^+^CD206^+^ M2-like macrophages. (**E**) Relative mRNA expression of M1-like macrophage markers. (**F**) Relative mRNA expression of M2-like macrophage markers. *n* = 6 per group. * *p* < 0.05, *** *p* < 0.001, **** *p* < 0.0001, ns, not significant. Error bars represent mean ± SEM.

To elucidate the regulatory effect of IL-38 on M1-like macrophage polarization, we first induced BMDMs with an M2-like phenotype from mouse bone marrow using M-CSF. Following IL-38 treatment, we observed a statistically significant attenuation of ox-LDL-induced M1-like polarization in these BMDMs (Figure 4A,B), whereas M2-like polarization remained unchanged with no statistically significant difference (Figure 4C,D). Consistently, the mRNA expression of M1-like macrophage-specific markers, including inducible nitric oxide synthase (*iNOS*), tumor necrosis factor-α (*TNF-α*), and *IL-6* were significantly downregulated in the IL-38-treated group (Figure 4E). In contrast, the mRNA expression levels of M2-like macrophage markers, including arginase-1 *(Arg-1)*, *IL-10*, and mannose receptor C type 1 *(Mrc-1)*, exhibited no significant alterations (Figure 4F). These results demonstrate that the primary role of IL-38 in the context of atherosclerosis is to modulate the inflammatory response by specifically inhibiting M1-like macrophage polarization, without affecting M2-like polarization.

### 3.5. IL-38 Acts on Multiple Inflammatory Pathways

**Figure 5:**
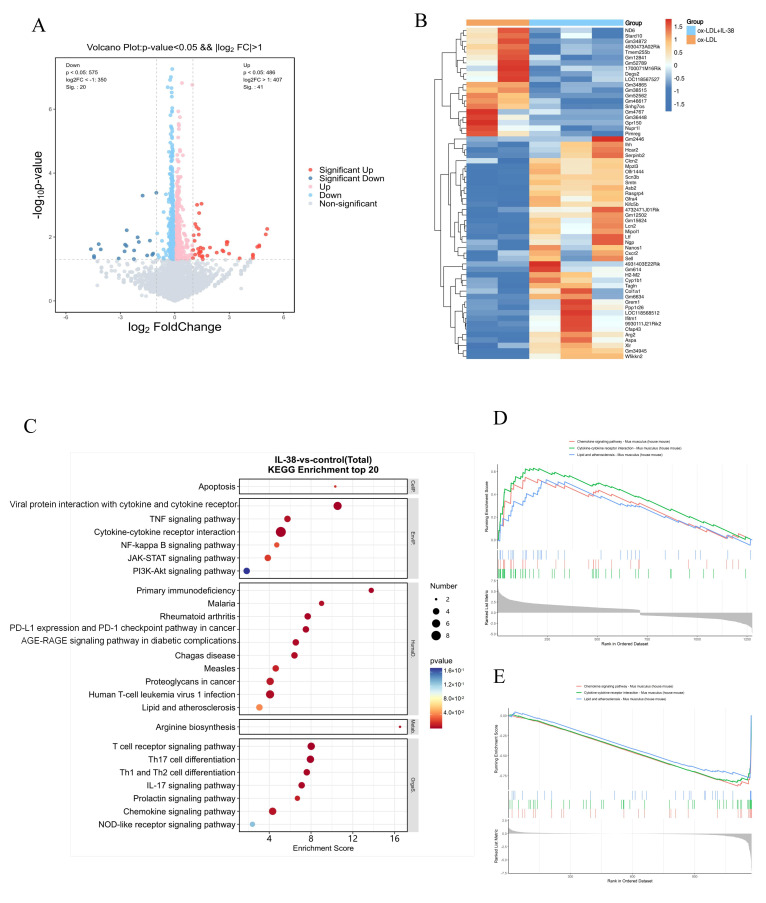
Analysis of gene regulation of IL-38 on macrophages. (**A**) Volcano plot showing the number and expression trends of DEGs in BMDMs treated with IL-38. (**B**) Heatmap of DEGs in BMDMs treated with IL-38. (**C**) KEGG enrichment analysis of DEGs, listing the top 20 significantly enriched signaling pathways (**D**) GSEA plots showing the significantly enriched gene sets in foam cells compared with non-foam cells. Data for foam/non-foam cell signature genes were retrieved from the public GEO database (accession ID: GSE116239). (**E**) GSEA plots showing the significantly enriched gene sets in macrophages co-stimulated with ox-LDL and IL-38.

Our findings revealed that IL-38 regulated the expression of 61 differentially expressed genes (DEGs) in ox-LDL-stimulated BMDMs, among which 41 were upregulated and 20 were downregulated (Figure 5A). Notably, a subset of these DEGs are well-characterized for their critical roles in macrophage-mediated inflammation (Figure 5B) and are involved in multiple inflammatory signaling pathways. In the present study, we therefore focused on validating whether IL-38 modulates the NF-κB signaling pathway and regulates macrophage apoptosis (Figure 5C). To further contextualize our results, we performed Gene Set Enrichment Analysis (GSEA) on RNA-seq data of macrophages isolated from mouse atherosclerotic plaques, which were retrieved from the public Gene Expression Omnibus (GEO) database (accession ID: GSE116239) [11]. Using this dataset, we first extracted the differential gene expression signature specific to foam cells in mouse atherosclerotic plaques (Figure 5D). We then compared this signature with our in-house RNA-seq data (Figure 5E). This comparison revealed that IL-38 significantly downregulated the expression of ox-LDL-induced, atherosclerosis-associated genes in macrophages.

### 3.6. IL-38 Inhibits NF-κB Pathway Activity

**Figure 6:**
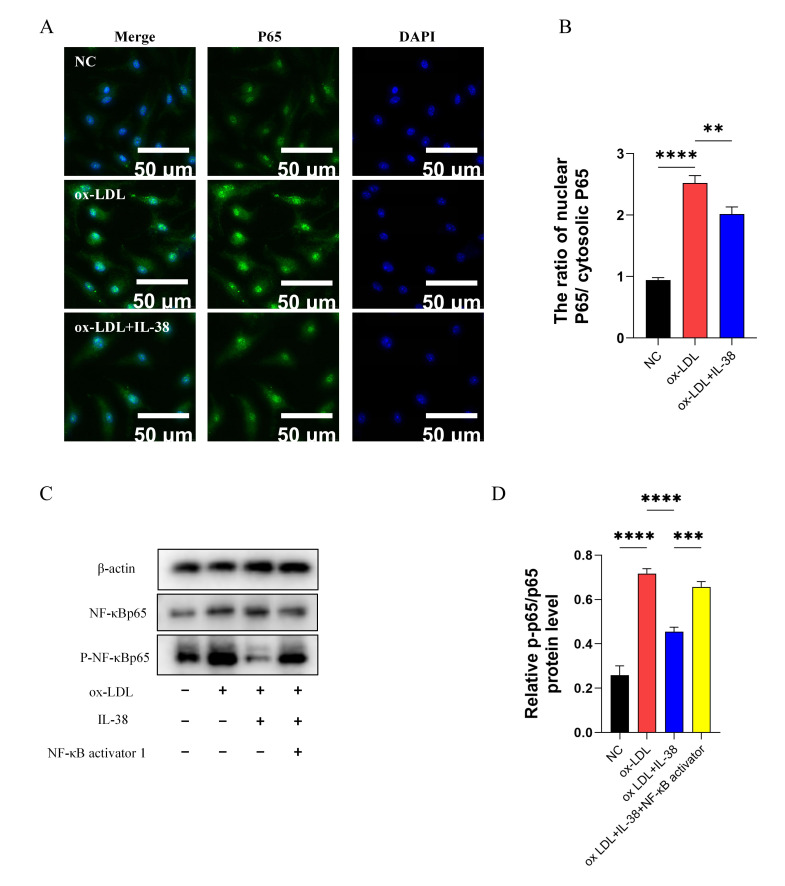
IL-38 inhibits NF-κB pathway activity in mouse bone marrow-derived macrophages (BMDMs). Experimental groups: (1) NC (negetive control): BMDMs without any stimulation; (2) ox-LDL group: BMDMs stimulated with 50 μg/mL ox-LDL for 48 h; (3) ox-LDL+IL-38 group: BMDMs stimulated with 50 μg/mL ox-LDL plus 50 ng/mL IL-38 for 48 h; (4) ox-LDL+IL-38+NF-κB activator 1 group: BMDMs pre-stimulated with 30 ng/mL NF-κB activator 1 for 6 h, followed by co-stimulation with 50 μg/mL ox-LDL and 50 ng/mL IL-38 for 48 h. (**A**) Immunofluorescence staining of p65 in BMDMs. (**B**) Quantitative analysis of p65 nuclear translocation. (**C**) Western blot of p-p65 and total p65 in BMDMs. (**D**) Quantitative analysis of p-p65/p65 protein expression ratio. *n* = 6 per group. ** *p* < 0.01, *** *p* < 0.001,**** *p* < 0.0001, ns, not significant. Error bars represent mean ± SEM.

To determine the regulatory effect of interleukin-38 (IL-38) on the NF-κB pathway in macrophages, we performed immunofluorescence staining of p65, the key functional subunit of the NF-κB pathway, in mouse BMDMs, followed by confocal microscopy analysis. Confocal images revealed that IL-38 treatment significantly reduced p65 nuclear translocation in BMDMs (Figure 6A,B). Furthermore, via Western blot analysis, we observed that IL-38 stimulation led to a statistically significant decrease in phosphorylated p65 (p-p65) expression in ox-LDL-stimulated BMDMs. To confirm that the inhibitory effect of IL-38 is specifically mediated by the NF-κB pathway, we pre-treated BMDMs with NF-κB activator 1 prior to IL-38 and ox-LDL stimulation. Notably, this pre-treatment almost completely abrogated the inhibitory effect of IL-38 on the NF-κB pathway (Figure 6C,D). These results provide compelling evidence that IL-38 specifically inhibits the activation of the NF-κB pathway in macrophages.

### 3.7. IL-38 Alleviates Macrophage Apoptosis

**Figure 7:**
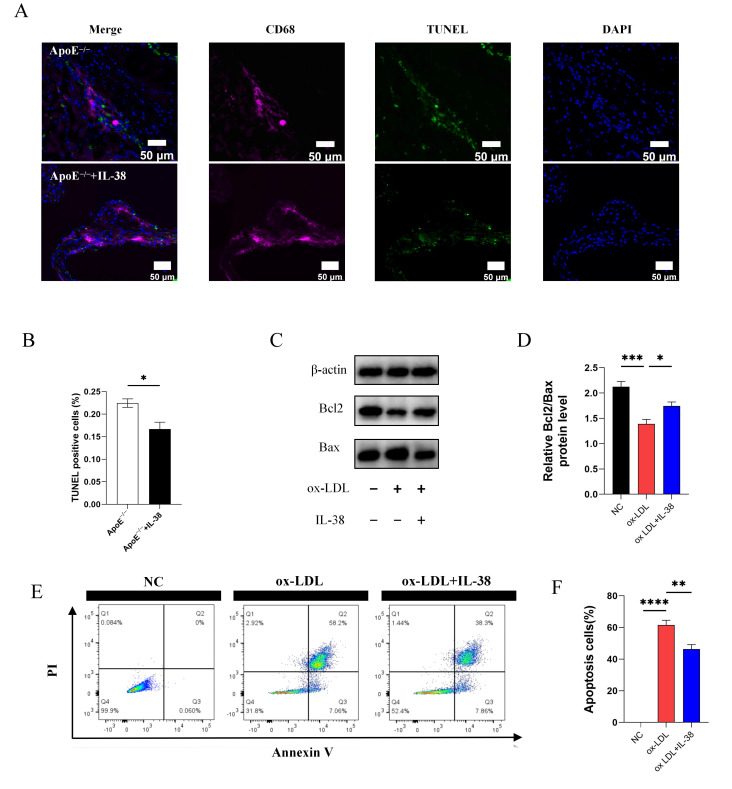
IL-38 affects apoptosis of mouse macrophages in atherosclerotic lesions and in vitro models. (**A**) CD68/TUNEL double immunofluorescence co-localization staining of mouse aortic roots. (**B**) Quantitative analysis of apoptotic macrophages. (**C**) Western blot of Bcl2 and Bax in mouse macrophages. (**D**) Quantitative analysis of the Bcl2/Bax protein expression ratio. (**E**) Flow cytometry dot plots of macrophage apoptosis. (**F**) Quantitative analysis of total macrophage apoptosis rate and the proportion of cells in each apoptotic stage. *n* = 6 per group. * *p* < 0.05, ** *p* < 0.01, *** *p* < 0.001, **** *p* < 0.0001. Error bars represent mean ± SEM.

To evaluate the effect of IL-38 on macrophage apoptosis in atherosclerotic lesions, we performed CD68/TUNEL double immunofluorescence co-localization staining on mouse aortic roots. Results showed that IL-38 exerted a statistically significant inhibitory effect on the proportion of apoptotic macrophages in mouse atherosclerotic plaques (Figure 7A,B). Concurrently, we observed a statistically significant increase in the protein expression ratio of Bcl2 to Bax in the IL-38 treatment group (Figure 7C,D). Bcl2 is an anti-apoptotic protein, and Bax is a pro-apoptotic protein, so this ratio elevation is a key indicator reflecting suppressed apoptotic signaling. Furthermore, a flow cytometry-based apoptosis assay revealed that IL-38 significantly reduced the apoptosis rate of ox-LDL-stimulated macrophages compared with that of the ox-LDL alone group, with a more pronounced reduction observed in the late apoptotic phase (Figure 7E,F). These findings collectively confirm that IL-38 exerts a protective effect by inhibiting macrophage apoptosis in the context of atherosclerosis.

## 4. Discussion

Oil Red O staining results showed that IL-38 significantly reduced atherosclerotic plaque formation in mice. Immunohistochemical analysis of plaques revealed a statistically significant reduction in the proportion of CD68^+^ macrophages, accompanied by decreased numbers of α-SMA^+^ smooth muscle cells and collagen fibers. Inflammatory macrophages contribute to foam cell formation and plaque instability by releasing inflammatory mediators. The underlying mechanisms include impaired efferocytosis, damage-associated molecular pattern-mediated inflammation, development of necrotic lipid cores, and thinning of the protective collagen cap, and ultimately lead to thrombosis and infarction [29]. Traditionally, reduced numbers of smooth muscle cells and collagen fibers in plaques are considered indicators of decreased plaque stability and increased rupture risk [2]. However, recent studies have shown that inhibiting the phenotypic switch of smooth muscle cells to synthetic vascular smooth muscle cells may enhance plaque stability [30]. These synthetic smooth muscle cells can express macrophage-like phenotypes and phagocytose cholesterol to form foam cells [31]. Two hypotheses have been proposed regarding IL-38’s effect on smooth muscle cell-mediated plaque stability. The first suggests that IL-38 reduces synthetic smooth muscle cell numbers to decrease foam cell formation. The second proposes IL-38 reduces smooth muscle cells in the fibrous cap to weaken plaque stability. Masson staining results, which showed reduced collagen fibers, provide stronger support for the second hypothesis. These opposing changes in plaque components mean the net effect of IL-38 on actual plaque rupture risk remains unresolved. Collectively, these data indicate that IL-38 reduces plaque macrophages, and this decrease in inflammatory macrophages modulates plaque composition by balancing inflammatory cell infiltration and structural component content [32].

Vcam and Icam are key cell adhesion molecules that promote monocyte accumulation in the arterial intima, a critical step in atherogenesis [33]. Inhibiting Vcam and Icam expression can reduce monocyte chemotaxis and delay atherosclerotic progression [34]. Notably, upon stimulation with IL-38, the mRNA expression levels of leukocyte adhesion molecules *Icam* and *Vcam*, as well as inflammatory factors including *Cxcl1*, *Cxcl3*, *Ccl2* and *Ccl12* in macrophages were decreased, which confirmed the inhibition of IL-38 on the inflammation of macrophages in the local plaque [35,36].

Macrophage subset dysfunction and abnormal death are two major mechanisms driving macrophage-mediated atherosclerosis [37]. M1-like macrophages are the primary effector cells of inflammatory responses [38,39]. Flow cytometry analysis of mouse splenic macrophages showed that IL-38 improved systemic inflammation. Immunofluorescence staining further revealed a significant reduction in CD68^+^CD86^+^ M1-like macrophages in local atherosclerotic plaques, with no significant effect on CD68^+^CD206^+^ M2-like macrophages. In vitro, M-CSF was used to induce M2-like macrophages [40]. When these macrophages were co-stimulated with ox-LDL and IL-38, we investigated the specific impact of IL-38 on M1-like polarization. IL-38 reduced the proportion of CD45^+^CD11b^+^F4/80^+^CD86^+^ M1-like macrophages [41], suggesting that IL-38 attenuates atherosclerosis by inhibiting M1-like macrophage polarization. This indicates that IL-38 can improve atherosclerotic progression by inhibiting macrophage M1-like polarization, thereby suppressing macrophage inflammation and foam cell formation [42,43,44].

The NF-κB pathway is a canonical regulator of inflammatory responses. Previous epigenetic studies have confirmed that inhibiting this pathway benefits atherosclerotic outcomes [43,44]. Additionally, NF-κB mediates M1-like macrophage polarization and apoptosis [13,42,45,46]. Therefore, among the multiple pathways identified by RNA-seq as potential regulators of macrophage polarization, we focused on the NF-κB pathway. In our study, we detected NF-κB-p65 nuclear translocation and protein phosphorylation in macrophage models treated with IL-38. We further verified the inhibitory effect of IL-38 on NF-κB pathway activation by using an NF-κB activator to reverse this effect.

In early atherosclerosis, macrophage apoptosis is considered harmless and may limit lesion cellularity to inhibit plaque growth. However, as atherosclerosis progresses, caspase-mediated cleavage of GSDME mediates membrane permeabilization and secondary necrosis [47]. Macrophages clear dead cells via efferocytosis to prevent secondary necrosis [48]. Impaired macrophage apoptosis disrupts this clearance function, leading to persistent apoptotic cells that eventually undergo secondary necrosis. This triggers an inflammatory cascade that exacerbates inflammation and expands the necrotic core [49]. Additionally, macrophages differentiated into foam cells release proinflammatory mediators to amplify the inflammatory microenvironment and accelerate atherosclerotic progression [29]. In our study, we used a multifaceted approach including immunofluorescence staining, TUNEL staining, flow cytometry, and Western blot analysis to demonstrate that IL-38 inhibits macrophage apoptosis in both animal and cellular models. This finding implies that IL-38 may mitigate atherosclerotic progression by reducing the accumulation of apoptotic macrophage debris and inflammatory mediators that otherwise promote plaque growth. Additionally, IL-38 may suppress foam cell formation by enhancing controlled macrophage efferocytosis [50,51]. Endoplasmic reticulum stress is a known driver of macrophage death in atherosclerosis [47], but its relationship with IL-38 has not been reported. The specific mechanism by which IL-38 regulates macrophage apoptosis remains to be clarified.

Although we have provided comprehensive evidence illustrating the mechanism by which IL-38 ameliorates atherosclerosis, our study still has certain limitations that need to be addressed in future research. Firstly, our research relies primarily on ApoE^−/−^ mice fed a Western diet and in vitro BMDMs stimulated with ox-LDL. The applicability of these findings to other atherosclerotic models or human clinical settings remains to be verified, as the pathological process of atherosclerosis in humans is more complex than that in animal models. Secondly, the current study focuses on the effects of exogenous IL-38, while the specific role and regulatory mechanism of endogenously expressed IL-38 in macrophages during atherosclerosis progression have not been explored. Thirdly, although we confirmed that IL-38 inhibits the NF-κB signaling pathway, the precise upstream receptors through which IL-38 exerts its effects on macrophages have not been validated. Currently, most studies believe that the main receptors of IL-38 are IL-36R, IL-1R1 and interleukin 1 receptor accessory protein like 1 (IL-1RAPL1) [52,53,54,55]. The interaction between IL-38 and these receptors requires further investigation. Additionally, while we observed that IL-38 regulates macrophage cholesterol metabolism by affecting the expression of ABCG1, ABCA1, and SR-A, the specific molecular mechanisms underlying this regulation and how IL-38 modulates these transporters to influence cholesterol homeostasis still requires further research. Finally, the fixed concentration, frequency, and intraperitoneal route of IL-38 administration limit the understanding of how alternative delivery strategies, dosages, or treatment timings might impact therapeutic outcomes. Nevertheless, our study provides novel insights into the anti-atherosclerotic effect of IL-38, and future research is needed to address these limitations and further clarify its clinical application potential.

The future development of anti-atherosclerotic therapies based on the beneficial mechanisms of IL-38 requires further investigation. While the study confirms that IL-38 inhibits the NF-κB pathway, suppresses M1-like macrophage polarization, and reduces macrophage apoptosis, additional research is needed to address three key unresolved mechanisms closely tied to its anti-atherosclerotic effect: for macrophage cholesterol metabolism, future work should focus on unraveling the upstream molecular basis of IL-38’s regulation of cholesterol transporters and their post-translational modifications, as well as validating crosstalk between IL-38 and lipid-sensing pathways to clarify how it integrates inflammatory regulation with cholesterol homeostasis, and performing functional cholesterol efflux assays to link transporter expression changes to actual lipid handling capacity in macrophages. For endoplasmic reticulum (ER) stress, a known driver of macrophage apoptosis in atherosclerosis, studies should detect core ER stress markers in IL-38-treated macrophages, use ER stress activators or inhibitors for rescue experiments to confirm if IL-38 alleviates apoptosis by suppressing ER stress, and identify which branch of the unfolded protein response it specifically targets. For efferocytosis, critical for resolving plaque inflammation and preventing necrotic core expansion, research should employ functional assays, detect key efferocytosis-related molecules, and use IL-38-overexpressing or receptor-knockout macrophages to verify if this process is directly mediated by IL-38. Beyond these mechanisms, additional research is needed to verify IL-38’s specific upstream receptors via direct binding assays or macrophage-specific receptor knockout models (targeting IL-36R, IL-1R1, or IL-1RAPL1), as well as to explore the role of endogenously expressed IL-38 in atherosclerotic progression. Therapeutically, optimizing IL-38’s delivery is essential: moving beyond the current intraperitoneal injection with a fixed dose to test macrophage-targeted liposomes, evaluating pharmacokinetic profiles to determine optimal dosages and half-life in atherosclerotic tissues, assessing long-term safety, combining IL-38 with existing agents such as statins to test synergistic effects on plaque regression, and developing modified IL-38 derivatives with improved stability and receptor specificity. Additionally, clarifying the mechanisms underlying discrepancies between IL-38-regulated cholesterol transporter mRNA and protein levels, and exploring crosstalk between IL-38 and other atherosclerosis-related processes, will further deepen understanding of its protective effects. Despite these remaining needs, IL-38’s role as a natural anti-inflammatory cytokine with multifaceted effects on macrophage function and plaque biology positions it as a promising candidate for translation into clinical therapies for atherosclerotic cardiovascular diseases.

## 5. Conclusions

**Figure 8:**
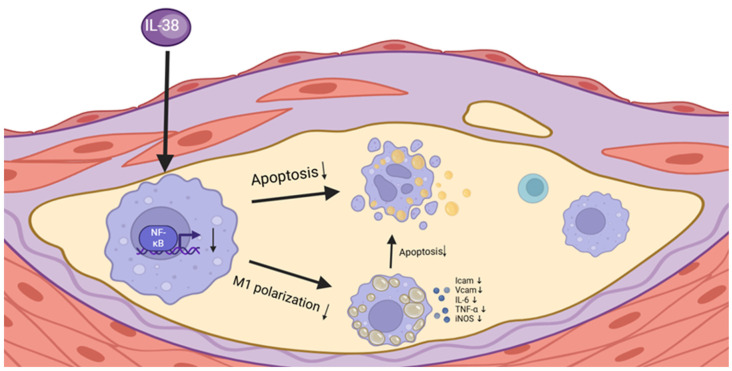
IL-38 ameliorates the process of atherosclerosis by inhibiting the polarization of macrophages to M1-like phenotype and apoptosis of macrophages. It also inhibits NF-κB pathway activity and alleviates inflammation during atherosclerosis.

Atherosclerosis is a chronic inflammatory disease that currently lacks effective targeted anti-inflammatory therapies. Given this, identifying relevant targets for anti-inflammatory intervention in atherosclerosis represents a critical strategy for the effective prevention and treatment of cardiovascular diseases. In recent years, IL-38 has emerged as an important anti-inflammatory cytokine that exerts protective effects in various chronic inflammatory conditions. However, its specific regulatory mechanism in atherosclerosis remains unclear. The present study demonstrates that IL-38 can ameliorate atherosclerotic pathogenesis in mice. Specifically, IL-38 delays atherosclerotic plaque formation by inhibiting macrophage-related inflammation and regulating the development of macrophage-derived foam cells. Additionally, IL-38 mitigates macrophage-driven inflammatory progression within atherosclerotic lesions through multiple mechanisms: suppressing M1-like macrophage polarization, inhibiting activation of the NF-κB signaling pathway, and reducing macrophage apoptosis (Figure 8). These findings highlight the potential of IL-38 as a novel therapeutic target for atherosclerosis, providing promising directions for developing anti-inflammatory strategies to address this prevalent cardiovascular condition.

## Data Availability

The original contributions presented in this study are included in the article/Supplementary Materials. Further inquiries can be directed to the corresponding authors.

## Supplementary Materials

The following supporting information can be downloaded at: https://www.mdpi.com/article/10.3390/biom15121741/s1, Figure S1: Purity determination of bone marrow-derived macrophages (BMDMs). Bone marrow-derived macrophages induced by macrophage colony stimulating factor were identified by flow cytometry as F4/80^+^CD11b^+^ macrophages. The content of macrophages in the samples exceeded 95%; Figure S2: (A) Plasma IL-38 concentration (pg/mL) in wild-type (WT) and ApoE^−/−^ mice. (B) Relative mRNA expression of IL-38 in mouse aorta. (C) Relative expression levels of TREM2 mRNA in macrophages stimulated by ox-LDL under different concentrations of IL-38. n = 6 per group. * *p* < 0.05, ns, not significant. Error bars represent mean ± SEM; Figure S3. (A) Scatter plot showing the correlation between plasma TG levels and three plaque-related positive areas: CD68^+^ macrophage area, lesion area, and necrotic core area. Spearman rank correlation analysis revealed no statistically significant linear correlation between TG and these plaque indicators (CD68^+^ area: R^2^ = 0.2515, *p* = 0.0967; lesion area: R^2^ = 0.2284, *p* = 0.1161; necrotic core area: R^2^ = 0.2574, *p* = 0.0922; all *p* > 0.05). (B) Scatter plot showing the correlation between plasma TC levels and the same plaque-related positive areas. Correlation analysis also showed no statistically significant linear correlation between TC and the plaque indicators (CD68^+^ area: R^2^ = 0.1460, *p* = 0.2203; lesion area: R^2^ = 0.1059, *p* = 0.3020; necrotic core area: R^2^ = 0.4249, *p* = 0.0216; all *p* > 0.05). (C) Scatter plot showing the correlation between plasma LDL-C levels and the same plaque-related positive areas. Correlation analysis also showed no statistically significant linear correlation between LDL-C and the plaque indicators (CD68^+^ area: R^2^ = 0.1769, *p* = 0.1642; lesion area: R^2^ = 0.0632, *p* = 0.5076; necrotic core area: R^2^ = 0.0104, *p* = 0.3130; all *p* > 0.05). Table S1: Primers used for real-time PCR of bone marrow-derived macrophages. Note: Primers were all obtained from PrimerBank. The primer design algorithm employed had been rigorously tested to ensure both PCR specificity and efficiency. Original Western blots.
