# Supplementary material for: Interleukin-38 Ameliorates Atherosclerosis by Inhibiting Macrophage M1-like Polarization and Apoptosis
Source: Biomolecules. 2025 Dec 16;15(12):1741. doi: 10.3390/biom15121741 (PMC12731223; doi:10.3390/biom15121741)

Supplementary data

Table S1. Primers used for real-time PCR of bone marrow-derived macrophages. Note: Primers were all obtained from PrimerBank. The primer design algorithm employed had been rigorously tested to ensure both PCR specificity and efficiency.

Gene	Forward Primer (5′-3′)	Reverse Primer (5′-3′)
ABCG1	CCGATGTCCCCTTTCAGATCA	AGCAGCGAACAGCACAAAAC
ABCA1	CCATGAAAGTGACACGCTGAC	TGCTGGCAAAGTACCATCTGAG
SR-A	AGCAAAGCAACAGGAGGACA	TTTTCACCTTGGGGTCCAGG
CD36	TTAATGGCACAGACGCAGCC	GGATTCTGGAGGGGTGATGC
Cxcl1	GCCACACTCAAGAATGGTCG	CTTGGGGACACCTTTTAGCA
Cxcl3	AAGATACTGAAGAGCGGCAAGTC	AGCAGGTAAAGACACATCCAGAC
Ccl2	CACTCACCTGCTGCTACTCA	GCTTGGTGACAAAAACTACAGC
Ccl12	GGCTGCTTGTGATTCTCCTGTAG	GGCTGCTTGTGATTCTCCTGTAG
Icam	GTGGGTCGAAGGTGGTTCTT	CCAGCCGAGGACCATACAG
Vcam	CTCTTACCTGTGCGCTGTGA	GGATCTTCAGGGAATGAGTAGACC
TREM2	GGTCAGCACGCACAACTTG	CGCAGCGTAATGGTGAGAGT
IL-38	AGCTTGGGATCTGCCTTCAG	CAGTATGGGTGGAGGGTTCAC

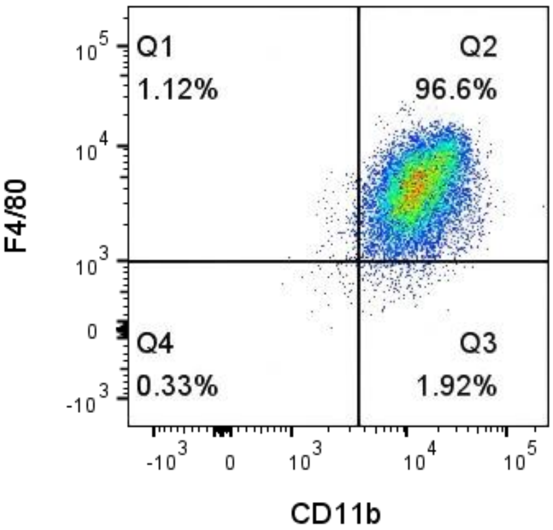

Figure S1. Purity determination of bone marrow-derived macrophages (BMDMs). Bone marrow-derived macrophages induced by macrophage colony stimulating factor were identified by flow cytometry as F4/80$^+$CD11b$^+$ macrophages. The content of macrophages in the samples exceeded 95%.

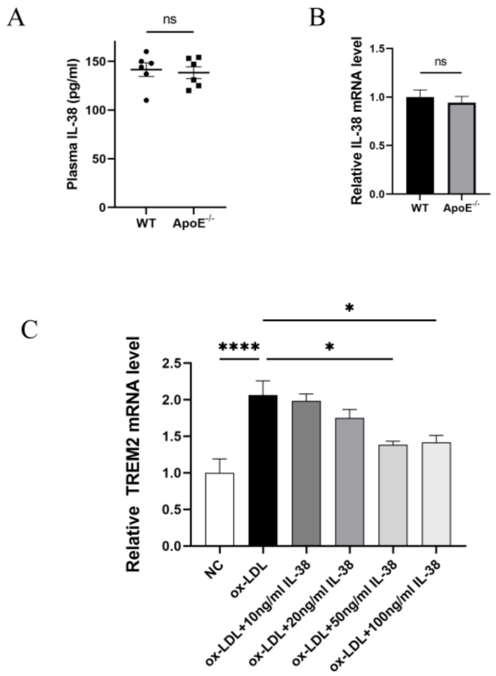

Figure S2. (A) Plasma IL-38 concentration (pg/ml) in wild-type (WT) andApoE$^{-/-}$ mice. (B) Relative mRNA expression of IL-38 in mouse aorta. (C) Relative expression levels of TREM2 mRNA in macrophages stimulated by ox-LDL under different concentrations of IL-38. n=6 per group. *p<0.05, ns, not significant. Error bars represent mean ± SEM.

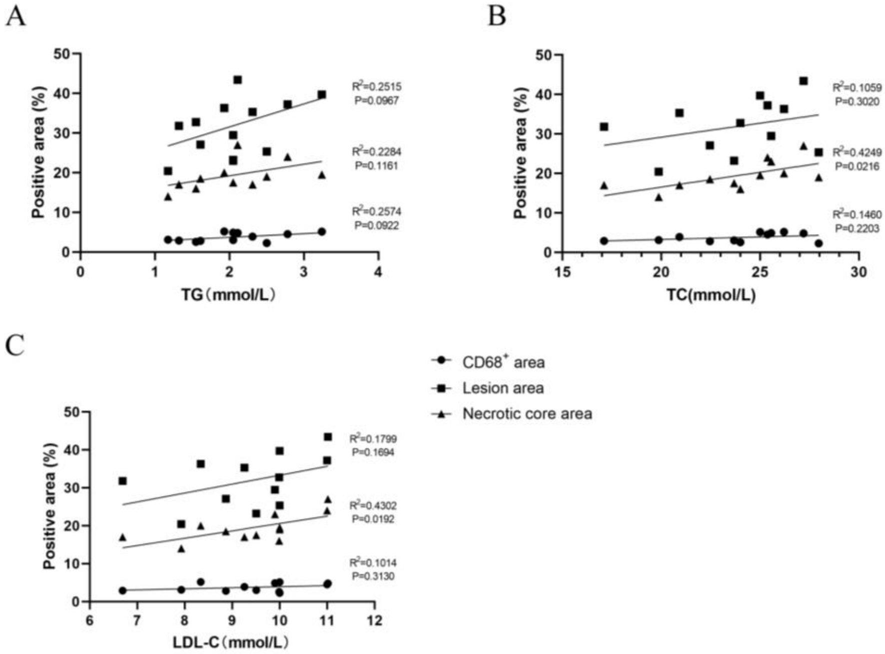

Figure S3. (A) Scatter plot showing the correlation between plasma TG levels and three plaque-related positive areas: $CD68^+$ macrophage area, lesion area , and necrotic core area. Spearman rank correlation analysis revealed no statistically significant linear correlation between TG and these plaque indicators ($CD68^+$ area: R^2=0.2515, P=0.0967; lesion area: R^2=0.2284, P=0.1161; necrotic core area: R^2=0.2574, P=0.0922; all P>0.05). (B) Scatter plot showing the correlation between plasma TC levels and the same plaque-related positive areas. Correlation analysis also showed no statistically significant linear correlation between TC and the plaque indicators ($CD68^+$ area: R^2=0.1460, P=0.2203; lesion area: R^2=0.1059, P=0.3020; necrotic core area: R^2=0.4249, P=0.0216; all P>0.05). (C) Scatter plot showing the correlation between plasma LDL-C levels and the same plaque-related positive areas. Correlation analysis also showed no statistically significant linear correlation between LDL-C and the plaque indicators ($CD68^+$ area: R^2=0.1769, P=0.1642; lesion area: R^2=0.0632, P=0.5076; necrotic core area: R^2=0.0104, P=0.3130; all P>0.05).

Original western blots.

Figure 2D

β-actin

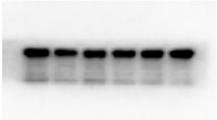

ABCG1

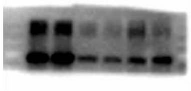

SR-A

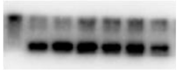

ABCA1

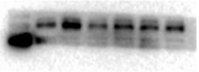

Figure 6C

β-actin

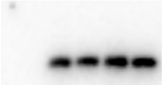

NF-κBp65

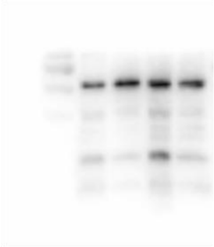

P-NF-κBp65

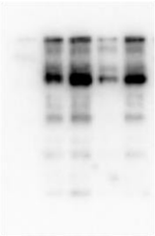

Figure 7C

β-actin

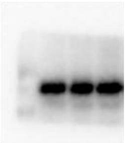

Bcl2

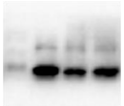

Bax

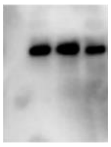

Supplement: Supplementary file 1 [file biomolecules-15-01741-s001.zip › biomolecules-3997345-supplementary.pdf]
